# A Study of the Relationship between Phthalate Exposure and the Occurrence of Adult Asthma in Taiwan

**DOI:** 10.3390/molecules28135230

**Published:** 2023-07-05

**Authors:** Tsai-Hui Duh, Chih-Jen Yang, Chien-Hung Lee, Ying-Chin Ko

**Affiliations:** 1 Department of Medicinal and Applied Chemistry, College of Life Science, Kaohsiung Medical University, Kaohsiung 80708, Taiwan; 2 Research Center for Precision Environmental Medicine, Kaohsiung Medical University, Kaohsiung 80708, Taiwan; cnhung@kmu.edu.tw; 3 Department of Medical Research, Kaohsiung Medical University Hospital, Kaohsiung Medical University, Kaohsiung 80708, Taiwan; 4 Division of Pulmonary and Critical Care Medicine, Department of Internal Medicine, Kaohsiung Medical University Hospital, Kaohsiung Medical University, Kaohsiung 80708, Taiwan; chjeya@kmu.edu.tw; 5 School of Post-Baccalaureate Medicine, College of Medicine, Kaohsiung Medical University, Kaohsiung 80708, Taiwan; 6 Department of Public Health, College of Health Science, Kaohsiung Medical University, Kaohsiung 80708, Taiwan; 7 Department of Medical Research, China Medical University Hospital, China Medical University, Taichung 40447, Taiwan; ycko0406@gmail.com; 8 Graduate Institute of Toxicology, College of Medicine, National Taiwan University, Taipei 10051, Taiwan

**Keywords:** adult asthma, phthalate exposure, phthalate metabolites

## Abstract

Although phthalate esters contribute to airway remodeling by increasing bronchial cells’ migration and proliferation, the relationship between human exposure to phthalates and asthma is not understood. We measured phthalate exposure in the human body and evaluated its effect on asthma. Asthma (*n* = 123) and asthma-free (*n* = 139) participants were, respectively, recruited from an asthma clinic and the community in Taiwan. The urine levels of six phthalate metabolites were determined by liquid chromatography tandem mass spectrometry. Compared with the controls, male asthma patients had higher means of mono-(2-ethylhexyl) phthalate (MEHP) (116.3 nmol/g), monobutyl phthalate (MBP) (850.3 nmol/g) and monoethyl phthalate (MEP) (965.8 nmol/g), and female patients had greater MBP (2902.4 nmol/g). Each 10-fold increase in the level of these phthalate metabolites was correspondingly associated with a 5.0-, 5.8-, 4.2- and 5.3-fold risk of contracting asthma. Male asthma patients were identified to have a higher proportion of MEHP exposure (32.5%) than the controls (25.3%). In asthma patients, an increase in urine MEHP levels and the total phthalate metabolite concentration were notably linked to increased risks of emergency room visits and being hospitalized. For the occurrence and acute clinical events of adult asthma, phthalate exposures and MEHP retention may contribute to higher risks of contracting this respiratory disorder.

## 1. Introduction

Exposure to various environmental factors can act as a trigger of and exacerbate asthma. Both indoor and outdoor allergens, irritants as well as cold temperatures can contribute to asthma exacerbation. Among household exposures, dust mites and cockroach allergens, along with the irritant effects of environmental tobacco smoke, play significant roles in asthma morbidity. Furthermore, occupational asthma stands out as the most prevalent occupational disease in industrialized countries. Allergens or irritants present in the work environment can either cause occupational asthma or worsen asthma symptoms in individuals with pre-existing asthma. It is worth noting that phthalates, also known as environmental hormones, have been implicated in various health concerns. However, only a limited number of studies have examined the association between asthma and phthalates. Therefore, further research is necessary to better understand the potential relationship between asthma and phthalate exposure [1].

Phthalic acid diesters, commonly called phthalates, are synthetic compounds that have been used as plasticizers for polyvinyl chloride (PVC) formulations since the 1930s. Phthalates are produced and used in cosmetics, food packaging, medicine coatings, lubricants, adhesives, toys, medical devices and building material [2,3]. Human exposure to phthalates can occur through oral, dermal or inhalation routes. The transmission paths of human exposure to phthalate are water, air, including skin and respiration, and food in particular. Food contamination can occur during production, processing, packaging and storage. The ubiquitous presence of phthalates in consumer products and the environment result in widespread exposure in the general population [2,4]. With a molecular structure similar to hormones, phthalates have the potential to disrupt the endocrine system, leading to health concerns regarding their detrimental impacts on development and the reproductive system. Over the past few decades, increased attention has been given to the potential risks associated with phthalate exposure. It is important to note that children are particularly vulnerable and sensitive to phthalates, particularly during the early stages of growth and development [5,6,7]. Therefore, phthalates are also called environmental hormones. Plasticizers have recently become a hot topic of discussion in Taiwan because various beverages and foodstuffs have been found to be laced with plasticizers by several unscrupulous manufacturers. Public concern about the potential health risks associated with exposure to plasticizers and related phthalates has therefore greatly increased. Di-(2-ethylhexyl) phthalate (DEHP) is the major plasticizer used in polyvinyl chloride (PVC) production. Dibutyl phthalate (DBP) and diethyl phthalate (DEP) are used in consumer and personal care products, such as cosmetics, deodorants and pharmaceutical coatings [8]. DEHP, benzylbutyl phthalate (BzBP) and DBP are common in many kinds of plastic products, including vinyl flooring, paint and other building materials, toys, plastic bags, gloves, shoes and imitation leather. Normally, phthalates are rapidly hydrolyzed to their monoesters and excreted in urine 12–48 h after exposure [9]. Some phthalates are further metabolized to their oxidative metabolites and/or glucuronidated, which increases their water solubility and facilitates urinary excretion [10]. Therefore, urinary phthalate metabolites have been used extensively as biomarkers of human exposure [11,12,13]. The first step in the metabolism of DEHP is a very rapid hydrolysis of DEHP to mono-(2-ethylhexyl) phthalate (MEHP), and MEHP is then catalyzed by unspecific lipases to secondary metabolites such as mono-(2-ethyl-5-hydroxyhexyl) phthalate (5-OH-MEHP) and mono-(2-ethyl-5-oxo-hexyl) phthalate (5-oxo-MEHP). The primary and secondary metabolites of DEHP have been used to predict exposure to DEHP. In addition, monobenzyl phthalate (MBzP) is a marker of BzBP exposure, monobutyl phthalate (MBP) is a marker mainly of DBP exposure and monoethyl phthalate (MEP) is a marker of diethyl phthalate (DEP) [14].

Several animal studies have shown that some phthalates possess endocrine disrupting effects, teratogenicity, developmental toxicity and adverse reproductive effects [11,12,15,16]. A variety of studies have been performed in mice to examine the influence of phthalates (delivered via various routes of exposure) on immune responses. One mouse inhalation experiment indicated that MEHP has the ability to modulate the immune response to exposure to an allergen [4,17,18]. Epidemiological data point to a possible correlation between phthalate exposure and asthma and airway diseases in children [19,20,21,22]. Several studies have found phthalate-induced enhancements of mast cell degranulation and eosinophilic infiltration, which are important aspects of the early inflammation phase [17,23,24]. Other investigations have found higher concentrations of phthalates, especially DEHP, in indoor dust in the homes of asthmatic children compared to non-asthmatic controls, indicating an association between concentrations of DEHP in indoor dust and wheezing among preschool children [17,25,26]. However, the lack of direct evidence of exposure to phthalate metabolites in these asthma patients limits the interpretation of this epidemiologic data [17]. Recent studies of human urine samples in industrialized countries have highlighted the large extent of the population exposed to various phthalates [11,12,16,27]. Therefore, we conducted this case–control study to measure the urine phthalate metabolite levels in adults with or without asthma to clarify the association between adult asthma and phthalate exposure.

## 2. Results

The demographic and clinical characteristics of the study participants, overall and stratified by sex, are shown in Table 1. Overall, age, body mass index (BMI) and urinary creatinine level were comparable between the asthma patients and community controls (all *p* > 0.05). A significantly higher proportion of asthma patients were cigarette smokers (31.7%) and had a lower lung function (61.8% with FEV1 < 80%) compared to the controls (16.6% and 20.9%, respectively). Within one year of the interview, 13.0% of the asthma patients had four or more asthma attacks, 24.4% were treated for asthma in an emergency department and 4.9% were hospitalized for asthma.

The average total concentrations of the phthalate metabolites, including MEHP, 5-OH-MEHP, 5-oxo-MEHP, MBzP, MBP and MEP, for male and female asthma patients were 2398.3 and 4197.4 nmol/g creatinine, respectively, and 1146.2 and 1664.2 nmol/g creatinine, respectively, for male and female controls (Table 2). Male patients with asthma had a significantly higher mean of MEHP (116.3 nmol/g), MBP (850.3 nmol/g) and MEP (965.8 nmol/g) than the male controls. Taking confounders into account, each 10-fold increase (a log10 unit increase) in the level of these three phthalate metabolites was associated with a 5.0-, 5.8- and 4.2-fold increased risk of contracting asthma, respectively. However, an elevated risk was only seen with the MBP metabolite level in women (2902.4 nmol/g, aOR = 5.3, 95% confidence interval (CI), 1.8–15.5). Compared to those with the lowest quartile concentration of total phthalate metabolites, male subjects with the highest quartile concentration had a 5.8-fold higher risk of asthma. A significantly linear trend for asthma risk in males was also noted (*p* = 0.002).

Table 3 shows the retention distribution for the three MEHP-related metabolites in the urinary samples. Among male subjects, a higher proportion of MEHP retention (32.5%) was identified in asthma patients compared to the controls (25.3%), whereas a greater percentage of 5-OH-MEHP retention (47.9%) was found in the controls compared to the asthma patients (42.8%). Because of the diverse rates of metabolism of MEHP, we further investigated the effect of urinary MEHP metabolism on the risk of asthma, using the ratio of 5-OH-MEHP to MEHP concentrations as an index (Table 4). Controlling for the level of 5-oxo-MEHP, each 10-fold increase in the ratio was related to a 0.2-fold decreased risk of asthma (in contrast, a 5-fold increase in risk for each 10-fold decrease in the ratio). While the rate of metabolism of MEHP remained constant, a 4.2-fold higher asthma risk was identified for each 10-fold increase in the urinary level of 5-oxo-MEHP.

The relationship between total phthalate exposure and clinical asthma events is presented in Table 5. Asthma patients who had a more severe clinical event within one year of the recruitment generally had a higher level of total phthalate metabolites (log-scale mean: 3.18, 3.27 and 3.41 nmol/g creatinine for four asthma attacks or more, emergency asthma treatment and hospitalization for asthma, respectively). Relative to the healthy controls (the base outcome), each 10-fold increase in the concentration of total phthalate metabolites was related to a 4.3-, 6.4- and 8.8-fold elevated risk of developing a more severe asthma event. A similar risk pattern was observed in male asthma patients. In addition, a higher urine level of MEHP (*p* = 0.021) and total concentration of the six phthalates (*p* = 0.045) are significantly associated with hospitalization due to asthma attack.

## 3. Discussion

To the best of our knowledge, this is the first study to demonstrate a clear association between phthalate exposure and the development of adult asthma. We found that asthma patients had a significantly higher level of total urinary phthalate metabolites than healthy individuals in both genders. In men, MEHP, MBP and MEP were associated with an increased risk of asthma; however, in women, the related metabolite was MBP. Taking the retention of metabolites in urine into account, patients with a lower metabolism rate of MEHP had a higher asthma risk. Further, clinical events due to asthma were significantly related to a high level of total phthalate exposure.

Asthma is a chronic inflammation of the lungs in which the airways are reversibly narrowed with typical symptoms of wheezing, shortness of breath, cough and chest tightness. Much of the increase in asthma’s prevalence is believed to be linked to a combination of genetic factors and changes in the environment affecting the immune system [18,28]. There is increasing evidence for the association of harmful health effects with exposure to phthalates, particularly DEHP, which has raised public concern and debate [19,26,29]. Phthalates have been shown to have adverse effects on airways and immunologic systems in several studies and a meta-analysis review [17]. The addition of DEHP to PVC for flexibility has become increasingly popular [2]. Therefore, heated PVC fumes may contribute to the development of asthma in adults. A previous report showed that MEHP, the major metabolite of DEHP, may induce prostaglandins (PG) PGD (2), 9α,11βPGF2 and PGF2α and thromboxanes in the lungs, thereby increasing the risk of inducing inflammation in the airways, which is a characteristic of asthma [24]. Further in vitro and animal model studies have shown that high levels of phthalates from PVC products can modulate the murine immune response to a co-allergen [17,19]. Glue et al. reported rapid histamine release after incubation of human peripheral blood mononuclear cells with DEHP or MEHP and an anti-IgE antibody co-stimulant [23].

Previous studies conducted on meat wrappers, hospital and office workers, firefighters and PVC processors found an association between PVC-related occupational exposure and higher risks of developing asthma, allergies or related respiratory effects [25,30,31,32,33,34,35,36,37]. Similar findings have been reported for the association between phthalate exposure and child asthma [19,20,26]. However, the lack of objective exposure assessment limits a clear interpretation of these epidemiologic data [17].

Several phthalate monoesters in urine can be detected in the general population, including MEHP, MBzP, MBP and MEP. These urinary phthalate metabolites can be used as biomarkers of human exposure to phthalates and serve as a simple method to evaluate phthalate exposure and clinical diseases [8,11,12,27]. Techniques using liquid chromatography coupled with tandem mass spectrometry (LC/MS/MS) provide rapid methods for measuring phthalate metabolites in urine. Meeker et al. showed urine MEHP to be inversely associated with testosterone, estradiol and free androgen index (FAI), indicating that this urinary phthalate metabolite was inversely associated with circulating steroid hormone levels in adult men [16]. Our study also used LC/MS/MS analysis to measure the urine phthalate metabolites to evaluate phthalate exposure and adult asthma. We found that urine phthalate metabolites, including MEHP, MBP, MEP and their sum, were all significantly associated with the occurrence of adult male asthma. In addition, a significantly higher concentration of MBP was observed in adult female asthma patients. Interestingly, women in both the control and asthma group had more phthalates exposure, such as MBP and MEP, than men in our study, indicating that women had contact with more phthalate-containing goods, possibly because phthalates are often used in personal care products such as nail polish, deodorant, perfume, hand lotion, shampoo and soap.

Our results show a strong association between exposure to phthalates and asthma. MEHP, the primary metabolite of DEHP, induced hypersensitivity to the methacholine-induced contraction of rat tracheal muscles in a previous study, and several epidemiological studies have also demonstrated a strong association between DEHP and adult asthma [17]. Both male and female asthma patients had significantly higher urine MBP, the metabolite of DBP, than controls in this study. This is compatible with our previous research which showed that exposure of human bronchial epithelial cells to DBP caused epithelial cells to produce the inflammatory cytokines interleukin 8 (IL-8) and RANTES, which subsequently induced bronchial smooth muscle cell proliferation and migration. This indicates that airway exposure to DBP will result in asthma and airway remodeling [38]. We also found that high urine MEP, the metabolite of DEP, was significantly associated with asthma in male patients. However, the association between DEP and asthma is still unclear and has not previously been documented. Further studies are necessary to clarify the pathogenesis.

A higher proportion of MEHP retention was identified in male asthma patients compared to the controls, whereas a greater percentage of 5-OH-MEHP retention, the metabolite of MEHP, was found in the controls compared to the asthma patients. This indicates that the metabolism rate of MEHP is diverse in women and men. It is possible that male adults with asthma have a low metabolism rate for MEHP, leading to a greater retention of MEHP and consequently triggering the occurrence of asthma.

This study demonstrated an association between urine phthalate metabolites and the occurrence of asthma, providing stronger evidence of the link between phthalate exposure and asthma. Additionally, within the asthma group, we observed that asthma patients with an IgE level > 100 mg/dL had significantly higher levels of urine MEHP compared to asthma patients with an IgE level < 100 mg/dL, suggesting that IgE plays a role in the pathogenesis of phthalate-exposure-related asthma. It is known that total plasma levels of IgE are frequently elevated during immune responses to allergens. Several studies have demonstrated an increase in emergency room visits and hospital admissions with acute asthma attacks in children who are sensitized and exposed to high levels of sensitizing allergens in their homes, and the association of allergen sensitization and high allergen exposure with the markers of asthma severity in children and adults [28].

Finally, our study showed that significantly higher levels of urine MEHP, the major metabolite of DEHP, significantly increased the risk of hospitalization. After adjusting for the covariates, a greater level of MEHP, MBzP and MBP was related to an elevated risk of asthma hospitalization. The above findings indicate that phthalate exposure may serve as a possible sensitizing allergen to asthma, leading to acute exacerbation and hospitalization in Taiwan.

A potential limitation of the present study is the measurement of urinary phthalate metabolites at a single point in time, as phthalates are rapidly metabolized and excreted. Consequently, the metabolite concentrations in urine only reflect exposure in the preceding 1 or 2 days [16]. Another limitation is the small number of patients, especially the small number of hospitalized asthma patients. These patients have been unaware of phthalate exposure for a long time.

## 4. Materials and Methods

### 4.1. Study Subjects

Adults with newly diagnosed asthma were recruited from the Division of Pulmonary and Critical Care Medicine, Kaohsiung Medical University Hospital and Kaohsiung Municipal Hsiao-Kang Hospital, in Kaohsiung city, Taiwan, from August 2009 to August 2011. Participants were diagnosed with asthma if they had symptoms such as episodic breathlessness, wheezing, cough and chest tightness and/or spirometry demonstrating an increase in forced expiratory volume in one second (FEV1) of at least 12% and at least 200 mL from the prebronchodilator value, or a 20% decrease in FEV1 after inhalation of methacholine according to the Global Initiative for Asthma (GINA) guidelines. Four pulmonologists were involved in the study and diagnosed patients according to the same criteria. A total of 123 adults (57 males and 66 females) aged from 20 to 75 years with asthma were diagnosed and agreed to participate in this study. The control participants were also recruited between August 2009 and August 2011, from a community-based health survey conducted at local health stations in four randomly selected regions representing the population of Kaohsiung city. All of the control participants were clinically confirmed to have not had asthma symptoms since their childhood. Basic characteristics of the controls were recorded, including their lung function. In total, 139 healthy adult controls (77 males and 62 females) without asthma, aged from 20 to 75 years, were included in the study. The institutional review boards at Kaohsiung Medical University Hospital reviewed and approved this study, and all participants signed written sheets of informed consent.

We checked serum total IgE levels of all the asthma patients, and collected information from questionnaires including age, gender, body mass index (BMI), ethnicity, history of tobacco smoking, medication use (inhaled steroids, inhaled bronchodilators or oral corticosteroids), onset age of asthma, clinical laboratory measures and FEV1 in both cases and controls. We also recorded the number of asthma attacks within a year, the number of visits to the emergency department and asthma-related hospitalization in the asthma group. Clinical data with regard to the time of asthma attack (1–3 and ≥4 times), emergency asthma treatment (no and yes) and hospitalization for asthma (no and yes) that had occurred within one year of subject recruitment were also collected.

### 4.2. Sample Collection

A 30–50 mL urine sample was collected in a 250 mL glass beaker and immediately transferred into a 60 mL Teflon-capped amber glass bottle for phthalate monoester and creatinine analysis. To prevent possible contamination of the urine samples, all glassware was washed twice with acetone and hexane, and then baked at 200 °C for 2 h in a clean oven.

### 4.3. Urinary Phthalate Metabolite Measurements

**Figure 1:**
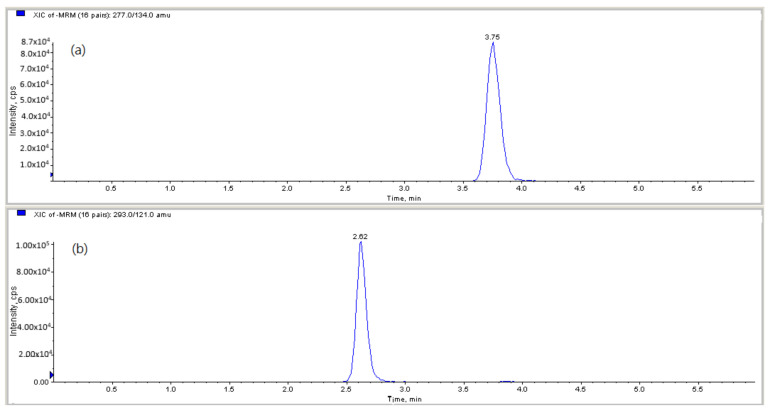

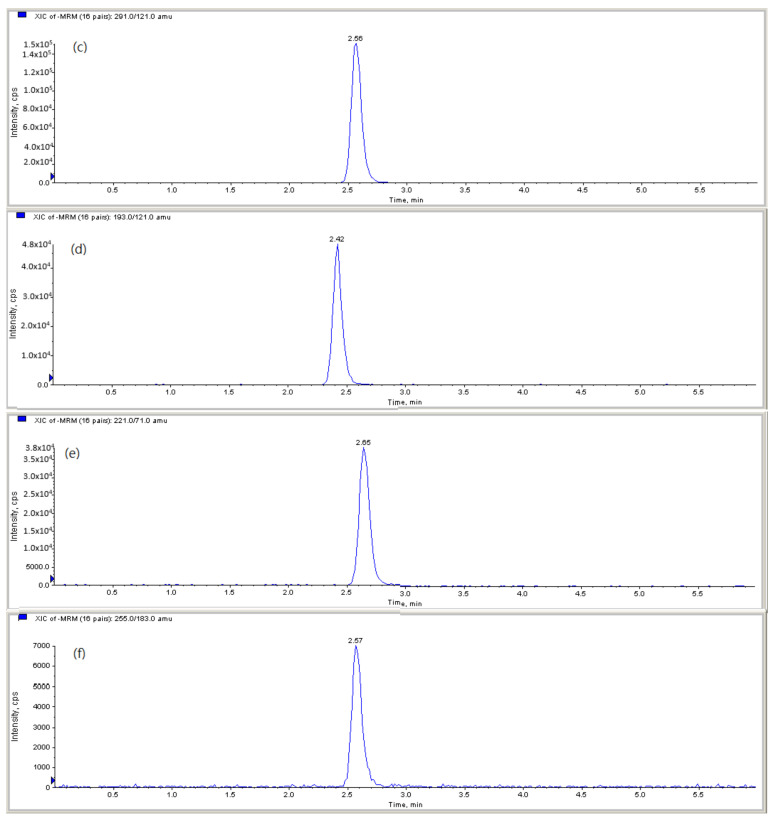
LC-MS-MS chromatograms of a standard mixture of phthalate metabolites spiked in urine: (**a**) MEHP, (**b**) 5-OH-MEHP, (**c**) 5-oxo-MEHP, (**d**) MEP, (**e**) MBP, (**f**) MBzP.

Mono-(2-ethylhexyl)) phthalate (MEHP), mono-(2-ethyl-5-hydroxyhexyl) phthalate (5-OH-MEHP), mono-(2-ethyl-5-oxo-hexyl) phthalate (5-oxo-MEHP), monobenzyl phthalate (MBzP), monobutyl phthalate (MBP) and monoethyl phthalate (MEP) were analyzed using on-line solid-phase extraction coupled with liquid chromatography tandem mass spectrometry (LC/MS/MS). A standard addition method [39] was used for the analysis of phthalate monoesters in human urine. LC-MS-MS chromatograms of a standard mixture of phthalate metabolites spiked in urine for MEHP, 5-OH-MEHP, 5-oxo-MEHP, MEP, MBP and MBzP are shown in Figure 1. Aliquots (400 μL) of urine sample in a series of Teflon-capped amber glass test tubes were separately added to an aliquot (50 μL) of a mixture of phthalate monoester standards at five levels of 0, 100, 250, 500 and 1000 ppb, each with a mixture of isotope phthalate monoesters (20 μL, 250 ppb) as internal standard, ammonium acetate (25 μL, 5M (pH 6.5)) and β-glucuronidase (5 μL, 200 U/mL). Each sample was incubated at 37 °C for 90 min to allow for the deglucuronidation of the phthalate metabolites. Then, the urine mixture was centrifuged and the supernatant was transferred into a glass screw-cap vial for subsequent on-line LC/MS/MS analysis [40]. The phthalate metabolites were elicited from a urinary matrix by on-line extraction on a trap column (LiChroCART 25–4 mm Rp-8 ADS; Merck) with a mixed solvent of 0.1% formic acid/H_2_O—acetonitrile (ACN) (95:5, *v*/*v*) at a flow rate of 1.0 mL/min for 10 min, chromatographically resolved by reversed-phase LC on an analytical column (ZORBAX Eclipse XDB-C18, 4.6 mm × 50 mm, 18 μm; Agilent, Santa Clara, USA) LC gradient program using an aqueous solvent A (0.1% formic acid/H_2_O) and an organic solvent B (acetonitrile) at a flow rate of 0.6 mL/min. This program was as follows: A:B (95:5%, 0–1.7 min) → A:B (95:5% → 5:95%, 1.7–2.5 min) →A:B (5:95%, 2.5–6.5 min) →A:B (5:95% → 95:5%, 6.5–7.5 min) →A:B (95:5%, 7.5–9.5 min). The effluent was detected by electrospray ionization tandem mass spectrometry (AB SCIEX API 4000) and quantified by isotope dilution. The detection limits of MEHP, 5-OH-MEHP, 5-oxo-MEHP, MBzP, MBP and MEP were 0.01, 0.01, 0.01, 0.1, 0.01 and 0.5 ng/mL, respectively. To assess accuracy, it is important to have a reference or known values to compare the measured values against. In this case, since there is no person in Taiwan who is not exposed to phthalates, it becomes challenging to directly measure the accuracy of the method by comparing the measured values with a known standard. Therefore, we examined the precision of the method in terms of the variances in the slopes and intercepts of the regression equations obtained from analyzing the phthalate monoesters in urine. Namely, five levels of a mixture of phthalate monoester standards were spiked to the urine samples with a fixed level of the internal standard for the standard addition analysis [39]. The regression equations were established with *y* for the peak area ratios of phthalate monoester to the internal standard and *x* for the concentration (ng/mL) of phthalate monoester. The relative standard deviations (RSDs) for the slope and intercept are all below 10.0%. To correct for urine dilution, we divided all phthalate metabolite concentrations by the urinary creatinine concentration, and used the resulting creatinine-corrected concentrations for our primary analysis.

### 4.4. Statistical Analysis

To take the effect of urinary dilution into account, all of the urinary concentrations for phthalate metabolites were adjusted for urinary creatinine. Since the concentration of urinary metabolites followed a log-normal distribution, all data were log10-transformed before statistical analysis. However, in order to reveal the actual levels of phthalate exposure, the untransformed data were also presented. Differences in log-scale mean and categorical distribution between asthma patients and controls were examined using *t*-tests and chi-squared tests. We employed logistic regression models to assess the relationship between phthalate metabolite levels (log-scale) and the risk of developing asthma. Age (continuous variable), FEV1 (<80% and ≥80%), cigarette smoking (no and yes) and gender, where appropriate, were considered as confounders, and were adjusted for in the multivariable regression models. The adjusted odds ratios (aORs) represented the relative odds of contracting asthma for each 10-fold increase in metabolite level. Because all 6 metabolites are phthalic acid diesters that are used as plasticizers in PVC formulations, we calculated the sum of the concentrations of the 6 metabolites to assess the overall effect of various phthalates on the development of asthma. Similar analysis strategies have been used in previous studies [41,42].

To study the metabolic conditions for urinary MEHP, we calculated the retention proportion of the three MEHP-related metabolites by dividing the urinary levels of MEHP, 5-OH-MEHP and 5-oxo-MEHP, respectively, by the total levels. Because the retention proportions of MEHP and 5-OH-MEHP were observed to be different between male asthma patients and controls (Table 3), we used the ratio of 5-OH-MEHP to MEHP concentrations as an index to represent the metabolized rate of MEHP, where a higher value indicates faster metabolism of MEHP. As clinical events with regard to the time of asthma attack (control, 1–3 and ≥4 times), emergency asthma treatment (control, no and yes) and hospitalization for asthma (control, no and yes) within one year of the recruitment were defined in three categories, polytomous logistic regression models were used to assess the association between asthma events and total phthalate exposure [43,44]. This type of logistic regression model allows for simultaneous comparisons of a categorically dependent outcome with more than two levels.

## 5. Conclusions

In conclusion, we successfully demonstrated that high levels of urine phthalate metabolites, including MEHP, MBP and MEP, were significantly associated with the occurrence of adult male asthma and significantly higher MBP levels were found in adult female asthma. The food-intake-related MEHP and its low elimination rate significantly contributed to the development of male asthma, while female asthma was related to MBP, the phthalate to which exposure can result from multiple pathways. In particular, in asthma patients, an increase in urine MEHP levels and the sum of the six phthalates studied were both significantly associated with the risk of being hospitalized. Taken together, we conclude that exposure to phthalate metabolites may play an important role in adult asthma in Taiwan, including occurrence and hospitalization.

## Figures and Tables

**Table 1:** Demographic and clinical characteristics of asthma patients and community controls.

Characteristics	Asthma			Control			*p* Value ^a^
	Male (*n* = 57)	Female (*n* = 66)	Total (*n* = 123)	Male (*n* = 77)	Female (*n* = 62)	Total (*n* = 139)	
Age (years), mean ± SD	46.0 ± 14.0	47.8 ± 14.7	47.0 ± 14.3	50.0 ± 15.5	47.1 ± 16.3	48.7 ± 15.9	0.357
Cigarette smoking							
No	27 (47.4%)	57 (86.4%)	84 (68.3%)	54 (70.1%)	62 (100.0)	116 (83.5%)	
Yes	30 (52.6%)	9 (13.6%)	39 (31.7%)	23 (29.9%)	0 (0.0%)	23 (16.6%)	0.004
FEV1							
FEV1 ≥ 80%	20 (35.1%)	27 (40.9%)	47 (38.2%)	58 (75.3%)	52 (83.9%)	110 (79.1%)	<0.001
FEV1 < 80%	37 (64.9%)	39 (59.1%)	76 (61.8%)	19 (24.7%)	10 (16.1%)	29 (20.9%)	
Body mass index (kg/m^2^), mean ± SD	26.3 ± 5.2	24.8 ± 4.5	25.5 ± 4.9	24.6 ± 3.3	24.1 ± 5.0	24.4 ± 4.1	0.058
Urinary creatinine (mg/dL), mean ± SD	165.1 ± 136.9	121.9 ± 111.7	141.9 ± 125.4	201.1 ± 138.5	105.9 ± 99.0	158.6 ± 131.0	0.296
**Clinical treatment within 1 year**							
Asthma attack							
1–3 times	49 (86.0%)	58 (87.9%)	107 (87.0%)				
≥4 times	8 (14.0%)	8 (12.1%)	16 (13.0%)				
Emergency asthma treatment							
No	43 (75.4%)	50 (75.8%)	93 (75.6%)				
Yes	14 (24.6%)	16 (24.2%)	30 (24.4%)				
Hospitalization for asthma							
No	53 (93.0%)	64 (97.0%)	117 (95.1%)				
Yes	4 (7.0%)	2 (3.0%)	6 (4.9%)				

^a^ *p* values for the difference between total asthma and controls were obtained by *t*-tests and chi-squared tests for continuous and categorical variables, respectively. Data are presented as number (percentage) unless otherwise indicated.

**Table 2:** Distribution of and difference in creatinine-corrected concentration of urinary phthalate metabolites (nmol/g) between asthma patients and community controls.

Gender/Metabolites	Arithmetic Scale, Mean ± SD		Log10 Scale, Mean ± SD		*p* for Mean Diff. ^a^	Cont. aOR ^b^	(95% CI)
	Asthma	Control	Asthma	Control			
Men							
MEHP	116.3 ± 198.1	46.2 ± 40.1	1.76 ± 0.48	1.52 ± 0.38	0.002	5.0	(1.8–14.2)
5-OH-MEHP	156.9 ± 241.3	112.6 ± 122.1	1.97 ± 0.77	1.85 ± 0.44	0.242	1.4	(0.7–2.8)
5-oxo-MEHP	90.8 ± 140.1	59.3 ± 56.5	1.67 ± 0.47	1.60 ± 0.42	0.301	1.6	(0.6–4.1)
MBzP	214.2 ± 427.0	159.5 ± 382.7	1.88 ± 0.77	1.95 ± 0.40	0.460	0.9	(0.5–1.8)
MBP	850.3 ± 1282.0	502.3 ± 1039.6	2.73 ± 0.40	2.45 ± 0.39	<0.001	5.8	(1.8–18.3)
MEP	965.8 ± 4334.2	266.3 ± 260.6	2.46 ± 0.49	2.23 ± 0.46	0.006	4.2	(1.6–11.1)
**Total metabolites ^c^**	**2398.3 ± 4802.4**	**1146.2 ± 1247.2**	**3.19 ± 0.58**	**2.94 ± 0.30**	**0.001**	**7.6**	**(1.9–29.4)**
<p25, *n* (%)			8 (14.0%)	19 (24.7%)		1.0	
p25–p74, *n* (%)			19 (33.3%)	39 (50.7%)		1.6	(0.5–5.2)
≥p75, *n* (%)			30 (52.6%)	19 (24.7%)	0.004	5.8	(1.7–19.2)
*p* for trend						0.002	
**Women**							
MEHP	101.8 ± 125.5	116.8 ±160.5	1.77 ± 0.47	1.81 ± 0.46	0.679	1.3	(0.5–3.3)
5-OH-MEHP	152.7 ± 354.7	101.6 ± 88.5	1.87 ± 0.50	1.82 ± 0.46	0.600	1.0	(0.4–2.5)
5-oxo-MEHP	86.1 ± 212.8	59.9 ± 60.4	1.63 ± 0.45	1.59 ± 0.45	0.599	1.0	(0.4–2.6)
MBzP	133.3 ± 139.4	168.3 ± 182.4	1.85 ± 0.64	2.05 ± 0.42	0.037	0.5	(0.2–1.1)
MBP	2902.4 ± 15673.6	726.7 ± 993.3	2.87 ± 0.47	2.63 ± 0.41	0.004	5.3	(1.8–15.5)
MEP	821.1 ± 2463.6	490.9 ± 600.4	2.56 ± 0.46	2.41 ± 0.53	0.086	1.6	(0.6–3.8)
**Total metabolites ^c^**	**4197.4 ± 16,588.0**	**1664.2 ± 1459.1**	**3.22 ± 0.41**	**3.09 ± 0.34**	**0.040**	**3.4**	**(0.9–12.2)**
<p25, *n* (%)			7 (10.6%)	15 (24.2%)		1.0	
p25–p74, *n* (%)			41 (62.1%)	32 (51.6%)		1.2	(0.3–5.1)
≥p75, *n* (%)			18 (27.3%)	15 (24.2%)	0.124	3.1	(0.8–12.0)
*p* for trend						0.029	

^a^ *p* value for unadjusted log-scale mean difference and chi-squared test. ^b^ Continuous adjusted odds ratio (aOR) associated with one-log10 change was adjusted for age, FEV1 and cigarette smoking. ^c^ The concentrations of the 6 phthalate metabolites are summed. The p25 (25th percentile) and p75 (75th percentile) were 2.11 and 2.46 for men, and 2.16 and 2.69 for women, respectively.

**Table 3:** The distribution of and differences in retention proportion (%) ^a^ of urinary DEHP metabolites among asthma patients and community controls, stratified by gender.

Metabolites	Men				Women			
	Asthma *n* = 57	Control *n* = 77	Diff. (%)	*p* for Diff.	Asthma *n* = 66	Control *n* = 62	Diff. (%)	*p* for Diff.
MEHP, %	32.5 ± 20.0	25.3 ± 19.7	7.2	0.040	35.3 ± 23.5	38.7 ± 25.8	−3.3	0.445
5-OH-MEHP, %	42.8 ± 14.1	47.9 ± 14.7	−5.1	0.044	41.3 ± 16.5	38.9 ± 17.6	2.4	0.436
5-oxo-MEHP, %	24.7 ± 8.4	26.8 ± 9.4	−1.6	0.123	23.4 ± 9.8	22.4 ± 11.2	1.0	0.595
Total metabolites	100.0	100.0			100.0	100.0		

^a^ Retention proportions are displayed as the percentage of specific MEHP-related metabolite concentration relative to the total metabolite concentration.

**Table 4:** The risk of rate of metabolized MEHP associated with the metabolized rate of MEHP and 5-oxo-MEHP.

	log10 Scale, Mean ± SD (nmol/g)		*p* for Mean Diff. ^a^	Cont. aOR ^b^	(95% CI)
	Asthma	Control			
**Men**					
5-OH-MEHP/MEHP	0.13 ± 0.49	0.32 ± 0.48	0.024	0.2	(0.1–0.5)
5-oxo-MEHP	1.67 ± 0.47	1.59 ± 0.42	0.301	4.2	(1.3–13.6)
**Women**					
5-OH-MEHP/MEHP	0.09 ± 0.61	0.02 ± 0.62	0.468	0.8	(0.4–1.8)
5-oxo-MEHP	1.63 ± 0.45	1.59 ± 0.45	0.599	1.2	(0.4–3.6)

^a^ *p* value for unadjusted log-scale mean difference. ^b^ Continuous adjusted odds ratio (aOR) associated with one-log10 change was adjusted for age, FEV1 and cigarette smoking, as well as the covariates in the table.

**Table 5:** Association of urinary total phthalate metabolite concentrations (nmol/g) with clinical events for asthma treatment within 1 year.

Clinical Factor	No. of Subject	Total Metabolites ^a^ log10 Scale, Mean ± SD	Cont. aOR ^b^	(95% CI)
**Overall**				
Controls	139	3.00 ± 0.33	1.0	(base outcome)
Asthma patients: clinical event				
**Asthma attack**				
1–3 times	107	3.21 ± 0.51	4.7	(1.9–11.3)
≥4 times	16	3.18 ± 0.36	4.3	(1.1–16.1)
**Emergency asthma treatment**				
No	93	3.19 ± 0.49	4.3	(1.8–10.4)
Yes	30	3.27 ± 0.49	6.4	(2.1–19.0)
**Hospitalization for asthma**				
No	117	3.20 ± 0.49	4.5	(1.9–10.8)
Yes	6	3.41 ± 0.51	8.8	(2.2–35.2)
**Male**				
Controls	77	2.94 ± 0.30	1.0	(base outcome)
Asthma patients: clinical event				
**Asthma attack**				
1–3 times	49	3.19 ± 0.60	7.5	(1.9–29.6)
≥4 times	8	3.15 ± 0.42	8.7	(1.3–58.7)
**Emergency asthma treatment**				
No	43	3.14 ± 0.59	7.2	(1.8–28.4)
Yes	14	3.33 ± 0.55	10.1	(2.0–50.5)
**Hospitalization for asthma**				
No	53	3.17 ± 0.30	7.1	(1.8–27.8)
Yes	4	3.44 ± 0.58	15.4	(2.2–105.1)
**Female**				
Controls	62	3.09 ± 0.34	1.0	(base outcome)
Asthma patients: clinical event				
**Asthma attack**				
1–3 times	58	3.23 ± 0.42	3.3	(0.9–12.2)
≥4 times	8	3.21 ± 0.31	3.8	(0.4–34.5)
**Emergency asthma treatment**				
No	50	3.23 ± 0.40	3.5	(0.9–12.9)
Yes	16	3.22 ± 0.44	3.0	(0.5–16.2)
**Hospitalization for asthma**				
No	64	3.22 ± 0.41	0.3	(0.1–1.1)
Yes	2	3.35 ± 0.35	2.6	(0.02–277.1)

^a^ Metabolites included MEHP, 5-OH-MEHP, 5-oxo-MEHP, MBzP, MBP and MEP. ^b^ Continuous adjusted odds ratio (aOR) associated with one-log10 change was adjusted for age, gender, FEV1 and cigarette smoking. Controls were referred to as the base outcome.

## Data Availability

The data presented in the study are available on request from the corresponding author.
